# The contribution of leading diseases and risk factors to excess losses of healthy life in eastern Europe: burden of disease study

**DOI:** 10.1186/1471-2458-5-116

**Published:** 2005-11-03

**Authors:** John W Powles, Witold Zatonski, Stephen Vander Hoorn, Majid Ezzati

**Affiliations:** 1 Department of Public Health and Primary Care, Institute of Public Health, Robinson Way, Cambridge CB2 2SR, UK; 2 Cancer Epidemiology and Prevention Division, M Slodowska-Curie Memorial Cancer Centre, ul W.K.Roentgena 5, 02-781 Warsaw, Poland; 3 Clinical Trials Research Unit, University of Auckland, Auckland, New Zealand; 4 Department of Population and International Health, Harvard School of Public Health, 665 Huntington Avenue, Boston MA 02115, USA

## Abstract

**Background:**

The East/West gradient in health across Europe has been described often, but not using metrics as comprehensive and comparable as those of the Global Burden of Disease 2000 and Comparative Risk Assessment studies.

**Methods:**

Comparisons are made across 3 epidemiological subregions of the WHO region for Europe – A (very low child and adult mortality), B (low child and low adult mortality) and C (low child and high adult mortality) – with populations in 2000 of 412, 218 and 243 millions respectively, and using the following measures: 1. Probabilities of death by sex and causal group across 7 age intervals; 2. Loss of healthy life (DALYs) to diseases and injuries per thousand population; 3. Loss of healthy life (DALYs) attributable to selected risk factors across 3 age ranges.

**Results:**

Absolute differences in mortality are most marked in males and in younger adults, and for deaths from vascular diseases and from injuries. Dominant contributions to east-west differences come from the nutritional/physiological group of risk factors (blood pressure, cholesterol concentration, body mass index, low fruit and vegetable consumption and inactivity) contributing to vascular disease and from the legal drugs – tobacco and alcohol.

**Conclusion:**

The main requirements for reducing excess health losses in the east of Europe are: 1) favorable shifts in all amenable vascular risk factors (irrespective of their current levels) by population-wide and personal measures; 2) intensified tobacco control; 3) reduced alcohol consumption and injury control strategies (for example, for road traffic injuries). Cost effective strategies are broadly known but local institutional support for them needs strengthening.

## Background

The evolving picture of East-West disparities in health indicators across Europe have been documented in terms of life expectancy [1] and premature mortality [2] and in terms of death rates for major contributing causes [3,4]. In addition to aggregate or partial mortality measures, a comparative analysis of the level and distribution of diseases and injuries, and their risk factors, is a valuable guide to strategies for improving health and for reducing cross-population health differentials. An important aspect of such comparative analyses is the use of a consistent and comparable metric of lost healthy life and the attribution of such losses either to diseases or injuries or to the risk factors for those diseases and injuries. The Global Burden of Disease project for the year 2000 (GBD2000) [5] and the associated Comparative Risk Assessment (CRA) Project [6], use common metrics and comparable methodology to address the burden of disease and injuries and their risk factors. We use the databases (some only recently available [6]) and published results from this study, to evaluate the nature and reasons for the health disparities across Europe. The results are presented for 3 epidemiologically-defined subregions of the WHO region for Europe.

In analyzing the causes of the marked differences in health levels across Europe, we have deliberately restricted our scope to the more proximal determinants ('risk factors'), because knowledge of their role is more secure and lends itself more readily to quantitative analysis.

## Methods

### Populations

Following the Burden of Disease protocols, the WHO region for Europe (which extends to Israel, Turkey and the former Soviet republics of central Asia) is divided into 3 'sub-regions' on the basis of child and adult mortality levels – Europe A (very low child; very low adult mortality) with a population in 2000 of 412 millions, Europe B (low child, low adult mortality) population 218 millions and Europe C (low child and high adult mortality) with a population of 243 millions. These 'subregions' are neither contiguous nor culturally homogeneous and correspond only approximately to western Europe, Eastern Europe and the successor states to the Soviet Union. Table 1 lists the countries within each subregion.

**Table 1:** Global Burden of Disease sub-regions in Europe

	**Mortality stratum**	**Countries**
A	Very low child; very low adult	Andorra, Austria, Belgium, Croatia, Czech Republic, Denmark, Finland, France, Germany, Greece, Iceland, Ireland, Israel, Italy, Luxembourg, Malta, Monaco, Netherlands, Norway, Portugal, San Marino, Slovenia, Spain, Sweden, Switzerland, United Kingdom
B	Low child, low adult	Albania, Armenia, Azerbaijan, Bosnia and Herzegovina, Bulgaria, Georgia, Kyrgyzstan, Poland, Romania, Slovakia, Tajikistan, The Former Yugoslav Republic of Macedonia, Turkey, Turkmenistan, Uzbekistan, Yugoslavia
C	Low child, high adult	Belarus, Estonia, Hungary, Kazakhstan, Latvia, Lithuania, Republic of Moldova, Russian Federation, Ukraine

### Mortality and burden of disease

Detailed methods for the Global Burden of Disease (GBD) 2000 project are described elsewhere [5]. In summary, for 14 epidemiological regions of the world, including 3 in Europe, GBD 2000 provides estimates of mortality and burden of disease for over 130 diseases and injuries. Mortality data are from national vital registration systems, reported annually to the World Health Organization. For countries in the European region with incomplete mortality data, or, more commonly, with substantial proportions of deaths allocated to non-specific codes [7], demographic techniques and epidemiological models are used to estimate mortality by age, sex, and cause. We calculated probabilites of death within age intervals from groups of causes using life table methods [8].

Burden of disease is expressed in disability-adjusted life years (DALYs), an aggregate measure of loss of healthy life to either premature mortality or to non-fatal illness or injury [9]. Inputs used to estimate losses of healthy life include estimates of disease incidence and/or prevalence, severity, and duration, generally from systematic reviews of disease-specific epidemiological literature or disease-specific registries (e.g. cancer registries). Flows of lost healthy life extending to future years are discounted and weighted by the age at which the lost healthy life would have been lived. The conceptual issues and sensitivity of results to these methodological details on the estimates of DALYs lost are described elsewhere [10].

### Risk factors

The methods and data sources for the Comparative Risk Assessment project are described elsewhere [6,11]. In summary, the contribution of a risk factor to disease or mortality relative to some alternative exposure scenario (i.e. population attributable fraction, PAF, defined as the proportional reduction in population disease or mortality that would occur if exposure to the risk factor were reduced to an alternative exposure scenario [12-14]) is given by the generalized "potential impact fraction" in Equation 1. For each risk factor – disease pair, the population attributable fraction is then multiplied by total deaths or burden of disease to estimate risk factor attributable mortality or burden of disease.



where

*RR*(*x*): relative risk at exposure level *x*

*P*(*x*): population distribution of exposure

*P'*(*x*): counterfactual distribution of exposure

*m*: maximum exposure level

The estimates of burden of disease and injuries due to risk factors in the CRA project are based on a counterfactual exposure distribution that would (within the limits of current knowledge and data) result in the lowest population risk, irrespective of whether attainable using current interventions or policies. This is referred to as the *theoretical-minimum-risk exposure distribution *[15,16]. The theoretical-minimum-risk exposure distribution was zero for risk factors for which zero exposure could be defined and reflected minimum risk (e.g. no smoking). For some risk factors, zero exposure was an inappropriate choice because of physical lower limits to exposure reduction (e.g. particles in ambient air). For physiological risk factors such as blood pressure, where lower values are associated with lower risk, the lowest values reliably associated with favourable health outcomes were used to define the theoretical-minimum-risk exposure distribution. Alcohol has benefits as well as harms depending on the disease under consideration and the patterns of alcohol consumption. A counterfactual exposure distribution of zero was still the default choice for alcohol, however, in some regions (e.g. EUR-A) effects on vascular disease were estimated as negative (protective) and combined with hazardous effects on other diseases to derive an estimated net effect [17]. For details of the risk factors considered see Table 2.

**Table 2:** 10 leading risk factors for the European region, exposure variables, theoretical minima, and contributions to total disease burden in the European region (source: Table 1 and Figure 1 in Ezzati *et al*. ^1^). See Table 1 in Ezzati *et al*. ^1 ^for disease outcomes and data sources.

**Risk Factor**	**Exposure Variable**	**Theoretical Minimum**	**Contribution to European disease burden (%GBD)**
High blood pressure	Level of systolic blood pressure	115 SD 6 mmHg	12.8%
Tobacco	Current levels of smoking impact ratio (indirect indicator of accumulated smoking risk based on excess lung cancer mortality); oral tobacco use prevalence	No tobacco use	12.3%
Alcohol	Current alcohol consumption volumes and patterns	No alcohol use ^b^	10.1%
High cholesterol	Level of total blood cholesterol	3.8 SD 1 mmol/l (147 SD 39 mg/dl)	8.7%
High body mass index (BMI)	Body mass index, BMI (height over weight squared)	21 SD 1 kg/m^2^	7.8%
Low fruit and vegetable intake	Fruit and vegetable intake per day	600 g (SD 50 g) intake per day for adults	4.4%
Physical inactivity	Three categories of inactive, insufficiently active (<2.5 hours per week of moderate-intensity activity, or less than 4000 KJ/week), and sufficiently active. Activity in discretionary-time, work, and transport considered	All having at least 2.5 hours per week of moderate-intensity activity or equivalent (400 KJ/week)	3.5%
Illicit drugs	Use of amphetamine, cocaine, heroin or other opioids and intravenous drug use	No illicit drug use	1.6%
Lead	Current blood lead levels	0.016 μg/dl blood lead levels ^c^	0.8%
Unsafe sex	Sex with an infected partner without any measures to prevent infection (represented as parameters of an HIV model)	No unsafe sex	0.7%

^a ^The resulting haemoglobin levels vary across regions and age-sex groups (from 11.66 g/dl in under-5 children in SEAR-D to >14.5 g/dl in adult males in developed countries) because the other risks for anaemia (e.g. malaria) vary.

^b ^Theoretical minimum for alcohol is zero, the global theoretical minimum. Specific sub-groups may have a non-zero theoretical minimum.

^c ^Theoretical minimum for lead is the blood lead levels expected at background exposure levels. Health effects were quantified for blood lead levels above 5 μg/dl where epidemiological studies have quantified hazards.

## Results

**Figure 1:**
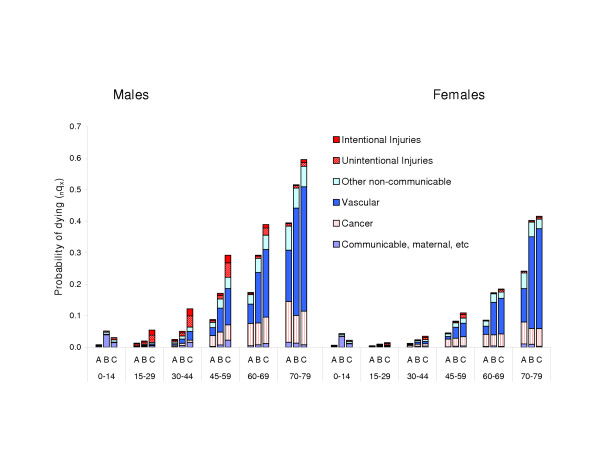
Probability of death within age intervals from 6 groups of causes by sex: Europe A, B and C, 2000.

The increase in mortality risks, by age group, from Europe A to B to C is illustrated in Figure 1. The most striking differences across the three subregions occur between ages 15 and 59. For example, for 30 year old males, the risk of death before reaching 45 in Europe B is twice that in Europe A, and in Europe C, nearly 5 times that in Europe A. For females, the relative mortality differences between Europe B and A are broadly similar, across all age intervals, to those for males, but mortality levels in Europe C are closer to those in Europe B than is the case for males. For example at ages 30 to 45 the risk of death in Europe C relative to Europe A is 2.8 fold for females compared to 5 fold for males. Beyond age 60, mortality risks in Europe B females are much closer to those in Europe C than to those in Europe A.

In males below age 60, gradients are present for all diseases but the biggest contributors to subregional differences are injuries (up to 8 fold differences between Europe C and A) and vascular diseases. Vascular diseases generally make the biggest absolute contributions to differences in mortality risks. In the age range 45 to 59, for example, risks of vascular death are 3 to 4 fold higher in B relative to A and 4 to 5 fold higher in C relative to A. Interestingly, *relative *differences are slightly greater in females (see Figure 1). Beyond age 60 in males, cancer and communicable diseases are not important contributors to mortality differences between subregions.

**Figure 2:**
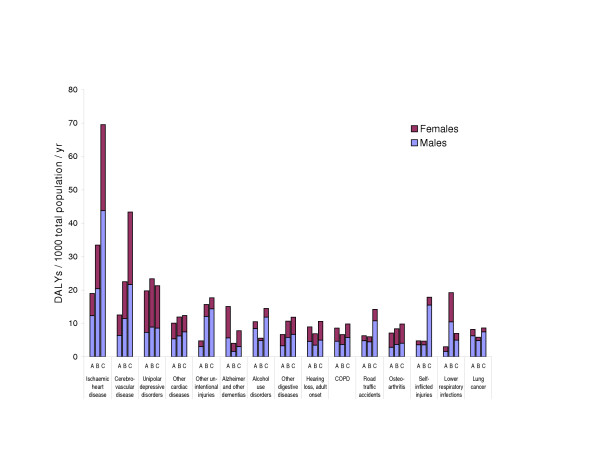
**Burden of disease due to 15 leading diseases or disease clusters in 2000: crude rates of DALYs per thousand population for Europe A, B and C, ordered by ranking for the combined European region**. Other cardiac diseases are those not classified as rheumatic, hypertensive, ischaemic or inflammatory. Other digestive diseases are those not classified as peptic ulcer, cirrhosis of the liver or appendicitis. Other unintentional injuries are those not classified as motor vehicle accidents, poisonings, falls, fires or drownings.

Figure 2 shows the estimated losses of healthy life from the 15 leading causes of disease burden in Europe as a whole. For several causes – unipolar depressive disorders, adult onset hearing loss, osteoarthritis and for two conditions primarily caused by smoking: chronic obstructive pulmonary disease and lung cancer – the magnitude of health loss (DALYs) is comparable across the 3 sub-regions. Age-specific mortality data show, however, that the rough equality of male burdens from lung cancer results from a balancing of higher risks in Europe B and C under the age of 60 with higher risks in A at ages over 60, a consequence of the historic lag of the smoking epidemic in Europe B and C compared to A.

The burdens experienced in Euro B and C in excess of those in Euro A come predominantly from 2 clusters of diseases: from vascular diseases (a gradient from 42 to 68 to 125 DALYs/thousand people/year from A to B to C when ischaemic heart disease, cerebrovascular disease and other cardiac diseases are combined for both sexes) and injuries (a gradient from 16 to 26 to 50 DALYs/thousand people/year from A to B to C when road traffic accidents, self-inflicted injuries and other unintentional injuries are combined for both sexes).

**Figure 3:**
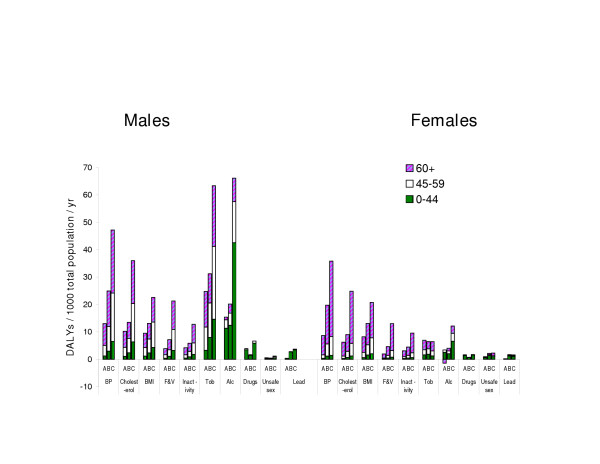
Burden of disease due to 10 leading risk factors: DALYs per thousand total population for Europe A, B and C, by sex and age group*. * DALYs are assigned to the age of death or of incidence (and not to the age at which the lost healthy life would have been lived)

The burden of disease attributable to leading risk factors in the 3 regions is shown in Figure 3. There is a substantial step down from the health losses attributed to the 7^th ^ranking cause, physical inactivity, to those attributed to the 8^th^, illicit drugs. The 7 leading risk factors can be divided into 2 clusters: a nutritional/physiological group contributing especially to risk of vascular disease – blood pressure, blood cholesterol concentration, body mass index, BMI (serving as an operational measure for excess adiposity), low fruit and vegetable consumption and physical inactivity. The second group comprises the major legal drugs – tobacco and alcohol.

The contributions of the legal drugs to sub-regional differences vary more by age and sex than do the contributions of the nutritional/physiological group of risk factors.

Much larger burdens are attributed to alcohol in males in Europe C, most strongly between ages 15 and 44, than in the other 2 subregions. Alcohol's contribution in Europe B is likely reduced by the proportion living in predominantly muslim states (Turkey and Uzbekistan alone account for more than a third of the population of Europe B and both are low alcohol consumers [18]).

Smoking contributes powerfully to the increases in disease burdens in males (but not in females) as one moves from Europe A to B to C. Further, the differences in male disease burdens attributed to smoking are relatively greater for streams of lost healthy life (DALYs) that begin in middle age and earlier: In the age range 45 to 59 the ratio of burdens attributable to smoking in Europe B to those in Europe A is around 2 fold and for C relative to A is around 3.5 fold. By contrast for males aged 60+ burdens attributable to smoking are comparable in B and A and less than 2 fold higher in C. Two factors are likely contributing to the greater differences in male burdens from smoking in middle rather than old age: i) very high tobacco consumption among younger men in in many countries in Europe B and most in Europe C, because of the historical lag in the decline in male smoking in Europe B and C relative to Europe A and; ii) the steeper gradient in vascular risks at younger ages, during which smoking acts as a more powerful multiplier than at higher ages.

The nutritional/physiological group of risk factors all show strong gradients in their attributed burdens across the 3 sub-regions, and assumedly account for much of the East-West gradient in premature vascular disease in both sexes (see also Discussion).

About half the DALYs attributed to smoking in Europe C are from vascular disease (because its multiplier effect is acting on very high background risks for vascular diseases), compared to just over a quarter in Europe A (where the background risks for vascular diseases have generally fallen markedly since the 1960s) [19]. Therefore, the magnitude of health losses from smoking, dominated by vascular diseases in the more disadvantaged European subregions, is strongly affected by the nutritional and physiological risk factors, much more so than vice versa. This multiplicative combination of multiple causes explains why such a large gradient exists in total burdens attributed to smoking whilst the gradient for lung cancer is more modest (Figure 2). Whilst the differences in burdens attributed to alcohol are much greater in younger adult males, the large differences in burdens attributed to the nutritional/physiological group of risk factors extend across all age-sex groups – making them responsible for a larger share of East-West health differences overall. At the same time, given the large hazards of alcohol on injuries and neuropsychiatric diseases, which are not affected by the other risks considered, measures to reduce health losses from alcohol will be important components of reducing East-West health differentials in Europe.

## Discussion

We have identified three risk factor/disease clusters as leading modifiable causes of excess health losses in Europe B and C: first, in order of importance, is the 'nutritional/physiological' group of risk factors (blood pressure, blood cholesterol concentration, body mass index, low fruit and vegetable consumption and physical inactivity) contributing primarily to very large absolute differences in vascular disease burdens; second, and largely because of its important multiplier effect on vascular risks, is tobacco; and third is the role of alcohol and other contributors to injuries as major sources of differences in health experiences of adult males.

This is the first report summarising differences in health levels and health determinants across Europe using results of the GBD2000 and CRA. The unique and central strength of the methods employed here is their ability to compare and rank the burdens of ill-health caused by different diseases and injuries and to similarly compare and rank the established causes of these diseases and injuries.

The metrics we have used have been developed to favour comprehensiveness and comparability – not ease of precise measurement. In particular, estimates of the 'years lived with disability' (YLD) component of the DALY are subject to significant uncertainty. This is because data for calculating time spent in non-fatal health states are less available than death registrations and because non-fatal health states require valuation before they can be incorporated into summary measures. In Europe as a whole, the 'years lived with disability' (YLD) component accounts for about 45% of DALYs lost. Findings as to the relative contributions of different diseases and injuries to subregional differences in levels of health are, however, robust to the uncertainty intrinsic to YLD estimates, because differentials are dominated by vascular diseases and injuries with large YLL component.

Despite 4 years of systematic data collection and analysis, leading sources of uncertainty for our findings on risk factors based on the CRA project are likely to be the estimated effects of alcohol on injury burden, neuropsychiatric conditions and vascular disease (mainly due to the heterogeneity of hazards across populations), and the estimated contributions of risk factors such as low fruit and vegetable consumption and physical inactivity to vascular risks (due to difficulties in defining and measuring exposure). Estimates for the hazards attributable to risk factors such as blood pressure, blood cholesterol concentration, body mass index and tobacco are likely to be more secure, drawing, as they do, on very large bodies of knowledge.

There is continuing scientific uncertainty about the ability of the classic risk factors such as those included in the CRA to adequately account for the high level [20] and temporal variation [3] in vascular disease rates, especially in Europe C. Several candidate risk factors, including neuro-humorally mediated exposures ('stress'), have been advanced to fill this explanatory gap [21,22]. CRA risk factors have, however, been deliberately limited to those 'for which there was good potential for satisfactory quantification of population exposure distributions and health effects using existing scientific evidence and available data ...' [6] (p xx) – criteria which excluded such candidates, as well as other nutritional risks that require valid data on dietary composition. Despite the potentially important role of these other factors, vascular risk in individuals is related similarly to the classic risk factors across cultures with widely differing risk factor levels [23,24] making them appropriate, albeit not exclusive, targets for public health policy cross-nationally, even if other, currently less well understood, influences are also contributing to differences in national levels and trends.

Because of multicausality and because multiple causes magnify each other's hazards (in a multiplicative way in standard epidemiological models), the contribution of any given risk factor to (absolute) disease burdens depends heavily on the other risks with which it is combining. For example, the burden of disease attributable to suboptimal cholesterol concentrations is over 3 times higher (in relation to population) in Europe C compared to Europe A (Figure 3) even though mean cholesterol concentrations are not higher in Europe C [25]. This combination of multiple risks implies that it is the absolute level of disease risks rather than the levels of individual risk factors that should determine the intensity of the public health response. Where absolute risks of vascular disease are high, as in Europe B and C, the leading public health priority must be *to reduce all amenable vascular risk factors irrespective of their starting levels *using complementary individual-level and population-wide strategies. An indication of the potential for such interventions is the decline over the past decade and a half in premature vascular deaths, most notably in females, in some former communist countries [26]. Recent rates of decline in countries such as Slovenia, Poland and the Czech Republic have been greater than in Europe A as a whole (unpublished observations).

The appropriate mix of 'high risk' and 'population' strategies for each country will depend on the resources available for medical care and on institutional capacities. Reducing risk in high risk individuals may be achieved by preventive counselling and changes in lifestyle and by 'chemoprevention' (eg long term medication to lower blood pressure and blood cholesterol concentrations). 'High risk' strategies relying on 'chemoprevention' will however, have much less effect on population disease burdens than 'population' strategies, unless large proportions of the adult population are placed on preventive medications [27].

Given the greater potential effectiveness of population approaches and the constraints on implementing high risk approaches, it will generally be preferable to start with population wide measures such as public education on the known causes of heart attack and stroke, and economic and regulatory approaches to help lower salt consumption and saturated fat consumption and to increase fruit and vegetable consumption, along with increased physical activity levels [28].

The second requirement, and potential, for health convergence across Europe is a decline in tobacco consumption in Europe B and C. Trends in some parts of Europe B are again encouraging, with all 8 new EU member states in Eastern Europe showing falling male lung cancer mortality in early middle age, since at least the mid 1990s [29]. The rise in smoking in young women has also been reversed in Poland [30]. Further East, in Russia, Belarus and the Ukraine there is sustained high smoking prevalence in males and rising prevalence in young females [31] – pointing to an urgent need to intensify control measures in those countries. The role of tobacco in levels and distributions of health across Europe is particularly important because economic (e.g. taxes) and regulatory (comprehensive advertising restrictions and bans on smoking in public places) measures have been shown to be highly effective in smoking reduction in many countries [32].

The third cluster to be addressed to reduce health inequalities across Europe is that of alcohol and injuries. The alcohol – injury cluster imposes particularly heavy burdens on Europe C males, especially under the age or 44 (Figure 3). Injuries account for over half the health loss atttributed to alcohol in Europe C males; and 43% of the health loss from injuries in this group is attributable to alcohol [17]. Recent economic analyses indicate (assuming generalisability of findings from studies done elsewhere) that an optimally cost effective strategy for averting overall harms to health from hazardous alcohol use in Europe C would be the combination of a 50% increase in tax, a ban on advertising and brief advice from physicians [33]. In addition to programmes to reduce alcohol consumption, effective means of reducing road traffic injuries are known, including the control of driving under the influence of alcohol which in Europe C males is estimated to account for 64% of traffic deaths.

The former communist countries dealt well with the public health challenges they faced in the period immediately following the second world war [34]. They failed however, to respond effectively to the more complex challenges posed by chronic disease and injury. These failures contributed to one of the gravest tragedies at the latter 20^th ^century – the loss of 2.5 – 3.0 million lives in Russia alone during the 1990s in excess of the losses expected at 1991 mortality levels [4]. The failure to develop public health infrastructures appropriate to the challenges faced, will have contributed substantially to these tragedies. Low rates of scientific publication in priority fields such as cardiovascular disease suggest serious under-investment in research of strategic importance to public health efforts: Medline indexed publication rates on cardiovascular diseases (per million population) are 7 fold lower in Europe B compared to Europe A and 7 fold lower again in Europe C (calculated from data in [35]). Low levels of relevant scientific activity will have provided little stimulus for the mass media to help raise public knowledge of chronic disease risks. Knowledge of established risk factors for vascular disease has been found to be low in Bulgarian [36] and Polish [37] populations. Remedial action is needed to address these infrastructural weaknesses throughout much of Europe B and all of Europe C if future public health endeavours are to be commensurate with the challenges faced.

The diseases and risk factors identified in this analysis also point to important data gaps and future research needs for using combinations of preventive and therapeutic interventions to reduce health disparities across Europe. These include: local analyses in the countries of Europe B and C to better define country-specific hazards for alcohol (including, for example, reasons for the very high mortality from liver cirrhosis in countries such as Hungary); better indicators and data on physical activity, diet and nutritional risks that can be used for identifying interventions; data on multi-risk correlation within countries (which is important for better quantification of hazards, for designing intervention packages for related risks and for within country equity); to develop scenarios for intervention packages and delivery options (including cost-effectiveness); to characterise local institutional strengths and weaknesses; and to assess risk reversibility.

## Competing interests

The author(s) declare that they have no competing interests.

## Authors' contributions

The scope and purpose of the paper was identified by JP, WZ and ME. The text was mainly written by JP and ME. SH provided analyses from the Global Burden of Disease databases. All reviewed and commented on the text.

## Pre-publication history

The pre-publication history for this paper can be accessed here:


